# COVID-19 Testing Experience in a Resource-Limited Setting: The Use of Existing Facilities in Public Health Emergency Management

**DOI:** 10.3389/fpubh.2021.675553

**Published:** 2021-06-14

**Authors:** Nega Assefa, Jemal Yousuf Hassen, Desalegn Admassu, Mussie Brhane, Mersen Deressa, Dadi Marami, Zelalem Teklemariam, Yadeta Dessie, Joseph Oundo

**Affiliations:** ^1^College of Health and Medical Sciences, Haramaya University, Harar, Ethiopia; ^2^Hararghe Health Research Partnerships, Haramaya University, Harar, Ethiopia; ^3^College of Agriculture and Environmental Sciences, Haramaya University, East Hararghe, Ethiopia

**Keywords:** coronavirus, COVID-19 testing, HHR, CAGED, Haramaya University, Ethiopia

## Abstract

**Introduction:** Coronavirus disease 2019 (COVID-19) is a public health emergency with little testing and treatment experiences at its occurrence. Diagnostic and treatment rapidly changed in the world including Ethiopia. Haramaya University has strived to change its diagnostic capacity using existing facilities in response to the national call to the pandemic.

**Objective:** This summary aims to detail experiences of setting up COVID-19 testing in Haramaya University laboratories, Eastern Ethiopia.

**Methods:** Desktop exercise was conducted to understand the start-up and implementations of COVID-19 testing in two Haramaya University laboratories, Hararghe Health Research Partnership and Campylobacter Genomics and Environmental Enteric Dysfunction laboratories. Communication, formats, guidelines, and standards were reviewed and summarized. Discussion with those involved in the start-up and implementation of the testing were also held. Ideas were summarized to learn the experiences the COVID-19 testing exercises.

**Lesson Learned:** This is a huge experience for Haramaya University to participate in the national call to increase the testing platform in the management of COVID19. Close work relationship with the public health authorities at all levels demonstrated the university's commitment to public service. The university has used the opportunity to advance its molecular testing capability by training its staff and students. The University has also contributed to the capacity development for laboratories in the surrounding areas of Harar, Somali, Oromia, and Dire Dawa. The pandemic has been an opportunity in harnessing existing resource for the benefit of the public during such times of dire needs to provide critical public health laboratory interventions.

## Introduction

Coronavirus disease 2019 (COVID-19) is an infectious disease caused by severe acute respiratory syndrome coronavirus 2 (SARS-CoV-2). The disease was first identified in December 2019 in Wuhan, the capital of China's Hubei province and has since spread globally, resulting in the ongoing 2019–2020 coronavirus pandemic (1). The outbreak of COVID-19 was declared a public health emergency of international concern on January 30, 2020, and thereafter declared a pandemic (2, 3). Following this, the World Health Organization has activated a worldwide surveillance, quarantine, testing, isolation, and treatment of positive cases.

Coronaviruses are a large family of enveloped, positive single-stranded RNA viruses that infect humans and a wide range of animals. These viruses were first described in 1966 (4). Experts of the International Committee on Taxonomy of Viruses termed it the SARS-CoV-2 virus as it is very similar to the one that caused the SARS outbreak (SARS-CoVs) (5).

The definitive diagnosis of active COVID-19 infection is based on the detection of either the spike protein or viral genes using real-time reverse transcription–quantitative polymerase chain reaction (RT-qPCR) tests. The genetic material test is the predominant test being implemented worldwide for detection, isolation, and treatment of COVID-19 cases (6). The test helps to detect carriers of the virus, which is fundamental in public health response efforts. It ensures the isolation of COVID-19 patients to prevent local spread and more broadly informs national response measures (7).

Other tests for immunoglobulin G (IgG) and IgM and point-of-care tests have been developed and used with varying sensitivities and specificities (8).

COVID-19 is a new phenomenon, and there was no preparation and institutional capacity for the testing in Ethiopia. Samples were initially shipped to South Africa as the Ethiopian Public Health Institute (EPHI) was in the process of putting infrastructure in place to do the testing in-country. Haramaya University laboratories have been and continue to be part of this effort. We present a summary of the establishment of testing platform, continuing testing, and challenges and lessons learned during the first 12 months of the continuing pandemic.

## Approaches

### Design

We conducted a desk review and discussions with individuals involved in the setup and conduct of the COVID-19 testing at the two laboratories in Haramaya University, Hararghe Health Research (HHR) Partnership and Campylobacter Genomics and Environmental Enteric Dysfunction (CAGED) laboratories under Haramaya University in Eastern Ethiopia. The summary focuses on the processes of start-up and implementation of COVID-19 testing between April 2020 and March 2021 in the two laboratories.

### Setting

**Figure 1:**
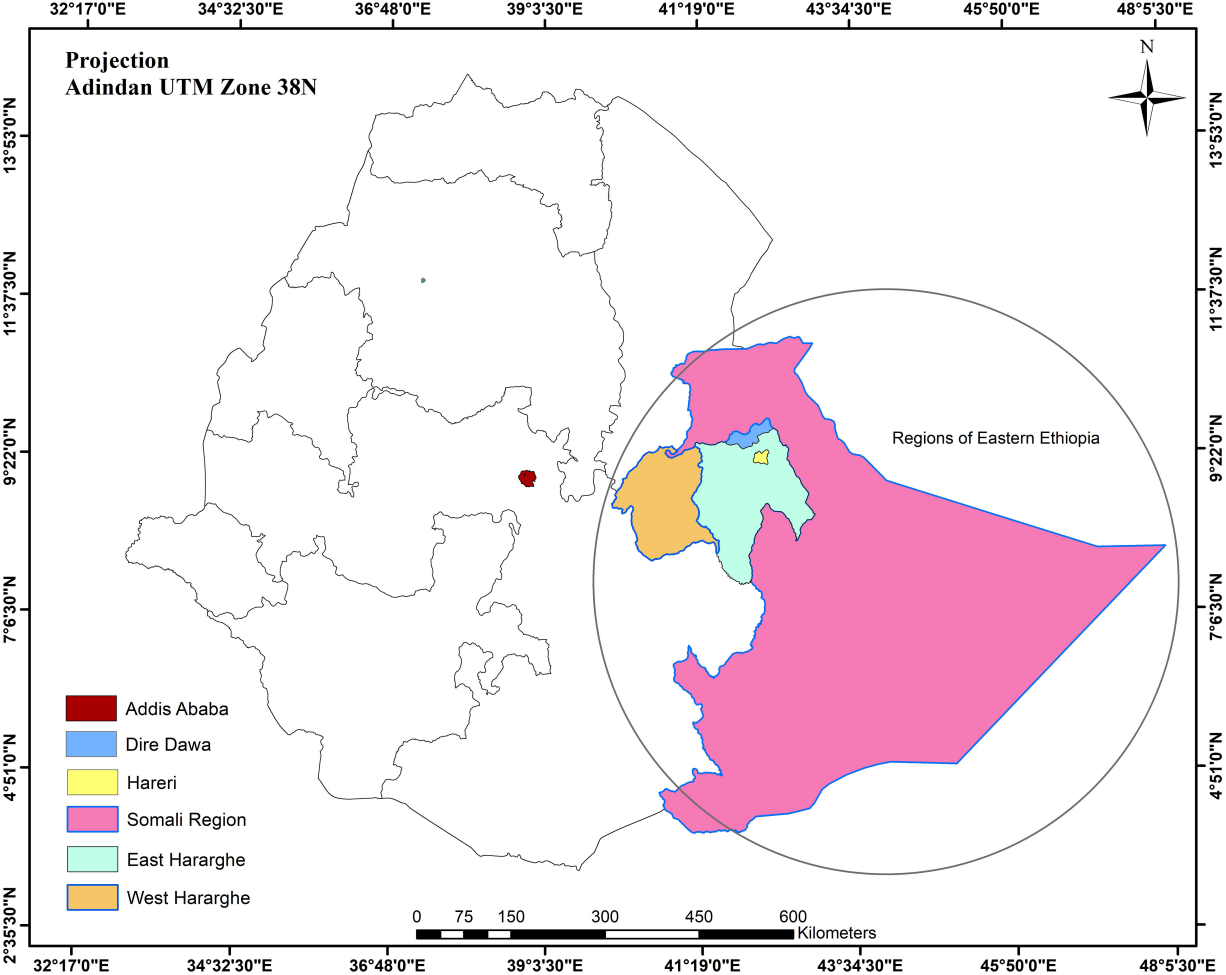
Ethiopia regions, Eastern Ethiopia, including Somali, Dire Dawa, Harari, and Eastern and Western Hararghe areas.

The HHR laboratory located in Harar campus in the premises of the College of Health and Medical Sciences, Haramaya University, is a collaborative effort between Haramaya University and London School of Hygiene and Tropical Medicine supported by the Bill & Melinda Gates Foundation as part of the Child Health and Mortality Prevention Surveillance effort to investigate the causes of stillbirth and death in children (9). The laboratory uses cutting-edge technologies to perform microbiological, molecular biology, and pathology tests (10). In addition to its regular research activities, the laboratory provides clinical diagnostic service support for cases requested by physicians from pediatric, gynecology, obstetric, and neonatal intensive care unit departments of Hiwot Fana Specialized University Teaching Hospital, Haramaya University, located in Harar (Figure 1). The other laboratory, CAGED, is a Bill & Melinda Gates Foundation–supported collaborative effort between University of Florida and Haramaya University set up to investigate the association of *Campylobacter* species exposure and childhood stunting in Haramaya district, Eastern Ethiopia (11, 12).

### Data Management

We reviewed communications between EPHI officers in Addis Ababa, Capital of Ethiopia, and Haramaya University administration, leading to the start-up of the testing service in the two laboratories. Continuing discussions focused on the training, testing setup, process of laboratory approvals for testing, and challenges encountered were held with the people involved in the testing chain.

## Key Summaries

### Initial Communications and COVID Testing Intentions

As the COVID-19 pandemic rapidly spread worldwide and it became a matter of when and not if the first case would appear in Ethiopia, it became critical to start preparing for the testing. Discussions were held with the Centers for Disease Control and Prevention Atlanta on the possibility of providing Taqman Array Cards capable of detecting SARS-CoV-2 using the Quant Studio 7 platform that we had in both laboratories. Meanwhile, the EPHI facilitated the start of testing using RT-qPCR on the same platform.

### Laboratory Assessment and Approval

The EPHI sent out prestart assessment forms to evaluate the laboratories for the presence of the required infrastructure. These included quality management systems, adequate and separate rooms for PCR and other laboratory work, reliable utilities, and ancillary PCR equipment and accessories. Other facilities assessed were sample storage and archiving capability, biosafety and biosecurity, and competent laboratory scientists. The HHR laboratory was deemed competent with no non-conformances and was designated by EPHI as one of the COVID-19 testing laboratories in Ethiopia. EPHI then assigned two senior laboratory experts to the HHR laboratory for 1 week to support with the setting up of the test workflow. This was also done for the CAGED laboratory in anticipation of a surge in testing, which would mean sending samples to that laboratory.

### Guidelines and Logistics

Standard protocol for specimen management, results communication, and the supply of test kits and other consumables were issued by the EPHI.

### Staff Training and Supply of Logistics

A total of six HHR and two CAGED laboratory staff were trained on all the pre-analytical, analytical, and post-analytical processes of the COVID-19 testing. The CAGED laboratory staff were deployed to perform the tests in the main campus laboratory. An additional five staff were later trained and added to the testing team when there was a surge in the test numbers. Logistical support initially for 100 tests from EPHI started with supply of extraction kit, centrifuge tube, film gown, micropipette tips, masks (N95 and surgical masks), gloves, face shields, PCR plates, adhesive PCR cover, falcon tube, absolute ethanol, head and shoe cover, and plastic apron. As the sample we received increased, the supply of logistics also increased to support more tests, and these have progressively been increased for the laboratory to have been able to test the maximum of 768 samples in a day.

### Start-Up of Tests

Initiation of sample testing was preceded by a letter from EPHI sent to health bureaus of Harari, Somali, and Oromia regions and Dire Dawa Administration toward the end of February, indicating that additional test facility has been established at Haramaya University and requesting these regions to send their samples to the said laboratory. The testing in the HHR laboratory was officially inaugurated by officials from Oromia and Harari region on April 12, 2020.

### Sample Reception

The nasopharyngeal and oropharyngeal samples in viral transport medium and packaged using triple packaging to meet International Air Transport Authority (IATA) regulations were transported to the laboratory and handled appropriately. Good laboratory practices were observed to ensure sample integrity and infection prevention and control for the people handling and testing the samples. The samples were logged into a laboratory samples register dedicated for COVID-19 and then received for processing by the laboratory staff.

### Conducting the Test

The samples were labeled and processed following the standard operating procedure earlier provided by the EPHI while observing strict quality control measures already in place in the laboratory. The laboratory takes part and receives SARS-CoV-2 external quality assurance panels from the United Kingdom External Quality Assurance Scheme UK NEQAS, which are processed alongside the patient test samples, with result scores being consistently above acceptable lower limits of 80% successful performance.

### Result Communication, Sample Archiving, and Disposal

The completed test results were reviewed by the tester, and one other reviewer for concurrence, and then the results were communicated to the Public Health Emergency Management sections of the respective regions from where the samples were collected and EPHI for the national daily COVID-19 updates. Aliquots of all samples testing positive for SARS-CoV-2 and 10% of negative tests are archived at negative 80°C freezers for further genomic analysis in the future. HHR laboratory is in the process of acquiring whole-genome sequencing capabilities to be able to perform this work in the future.

### Support for Regional Laboratories

The Haramaya University laboratories, while receiving and testing samples from the designated regions, also provided support to the Somali, Harari, and Dire Dawa regional laboratories and Oromia regional state by building capacity for these laboratories to be able to also test these samples. The Haramaya University provided a PCR machine to the Dire Dawa regional laboratory to be able to set up its testing facility. Currently, all these regional laboratories are competently performing the SARS-CoV-2 testing for samples received from regions and zones. Staff have been deployed to help with knowledge and skill transfer to the personnel working in their respective places.

### Tests Done so Far

Table 1 shows the areas Eastern Ethiopia and numbers of samples received from the respective places. There was a significant surge in the performance of the test during August 7 through September 8, 2020, due to ComBAT (Community-Based Testing) campaign set by the Ministry of Health and EPHI to evaluate the level of community level transmission.

**Table 1:** COVID-19 tests conducted during April 2020–March 2021, HHR, and CAGED Laboratories, Haramaya University, Eastern Ethiopia.

**Samples received from**	**No**.	**%**
Harari region	5,025	16.6
Somali region	4,543	15.0
Dire Dawa Administration	3,295	10.9
East/West Hararghe zones	17,362	57.5
Total	30,225	100

## Lessons Learned

- The COVID-19 pandemic has created an opportunity for collaborative work and support mechanisms between researchers, public health authorities, and universities to overcome a public health emergency.
- The effort has created an opportunity for faster, easier, and quality test result in the management of suspected COVID-19 cases and those in quarantine and isolation.
- Even though most of the equipment needed for the testing is available in our facilities, they were not set up for quick and rapid emergency public health response. This is a good lesson for other diseases of both local and international health concerns.
- The setting up and running of advanced scientific laboratories in the universities require large initial resource outlay; however, it has been demonstrated that the collaborative effort to setup HHR and CAGED laboratories have been a good investment. This is a critical lesson for researchers, colleges, and universities to invest time and resource in cultivating the culture of creating collaborative platforms with various agencies in the advancement and practices of sciences and public health responses.
- Resource mapping is another area to focus on. At the start of the pandemic, Ethiopia was sending samples to South Africa, and the turnaround time for results reception was days. This was due to lack of a single laboratory in the country set to do the COVID test. Currently, the country has 54 such facilities in public and private health facilities, universities, and research laboratories located in different part of the country all performing the testing. This is due to scoping activities throughout the country to learn what the country has and the gaps. Similar actions should continue to support the research and diagnostic capacity of facilities.
- Using such an opportunity to train staff and students cannot be overemphasized. The emergency has created an option for training staff from the college, hospital, and clinic at the main campus within our facilities. It also provided opportunities for medical microbiology students at the college to get real-time experience at the facility.

## Discussion

Public health emergencies need concerted efforts by all stakeholders working together collaboratively to combat the problems for a good outcome. These situations are also good learning opportunities and facilitating new knowledge and advancement of sciences and medical knowledge. COVID-19 pandemic provide just such an opportunity, which caught the attention of not only the public but also decision-makers at all levels of governments and international agencies worldwide to work together for a good outcome (13, 14).

Alongside the preparations to test and treat patients, the pandemic also created an opportunity for the various actors in the different sectors to pull together in a concerted way and allocate resources to respond to the pandemic. This can be taken as a good lesson in the response against other disease including tuberculosis, malnutrition, mental health, and the rising chronic diseases (15–17).

The experience with this pandemic has exposed the basic nature of most of the laboratory facilities in the universities and health sectors in general, not only in Ethiopia but also in Africa. There is an acute lack of state-of-the-art laboratory facilities that are able to be put to emergency use to provide emergency support in outbreaks of public health significance such as the ongoing COVID-19 pandemic. As a result, this created a vacuum of professionals and equipment as special tests are required in certain disease conditions. This experience should act as an impetus for the government of Ethiopia to act in advancing teaching and research laboratories in universities and other research facilities. A lesson of cultivating a culture of collaborative platform with various agencies in good times in the advancement of sciences and medical practice is necessary to lend a hand at times of emergency (18). Although this is a public health emergency, opportunities have enabled the staff, and students learn new techniques of molecular testing (14). The how of vaccines and drug development demonstrated how challenges turn out to be opportunities for sciences and medicine (19, 20). The experiences shared in this report are those of the Haramaya University and do not necessarily demonstrate the experiences of other universities and institutions in Ethiopia.

## Data Availability Statement

The data analyzed in this study is subject to the following licenses/restrictions: data related to this article can be sought from the first author. Requests to access these datasets should be directed to Nega Assefa, negaassefa@yahoo.com.

## Ethics Statement

Ethical approval for this study and written informed consent from the participants of the study were not required in accordance with local legislation and national guidelines.

## Author Contributions

NA, JH, JO, MB, MD, ZT, and YD facilitated the setup of the lab. MB, MD, DM, ZT, and DA are involved in the testing process. NA and YD drafted the paper. NA, JH, ZT, JO, MB, MD, DM, DA, and YD edited and approved for submission. All authors contributed to the article and approved the submitted version.

## Conflict of Interest

The authors declare that the research was conducted in the absence of any commercial or financial relationships that could be construed as a potential conflict of interest.
